# Optimal Defense Strategies in an Idealized Microbial Food Web under Trade-Off between Competition and Defense

**DOI:** 10.1371/journal.pone.0101415

**Published:** 2014-07-07

**Authors:** Selina Våge, Julia E. Storesund, Jarl Giske, T. Frede Thingstad

**Affiliations:** Hjort Centre for Marine Ecosystem Dynamics, Department of Biology, University of Bergen, Bergen, Norway; Uppsala University, Sweden

## Abstract

Trophic mechanisms that can generate biodiversity in food webs include bottom-up (growth rate regulating) and top-down (biomass regulating) factors. The top-down control has traditionally been analyzed using the concepts of “Keystone Predation” (KP) and “Killing-the-Winner” (KtW), predominately occuring in discussions of macro- and micro-biological ecology, respectively. Here we combine the classical diamond-shaped food web structure frequently discussed in KP analyses and the KtW concept by introducing a defense strategist capable of partial defense. A formalized description of a trade-off between the defense-strategist's competitive and defensive ability is included. The analysis reveals a complex topology of the steady state solution with strong relationships between food web structure and the combination of trade-off, defense strategy and the system's nutrient content. Among the results is a difference in defense strategies corresponding to maximum biomass, production, or net growth rate of invading individuals. The analysis thus summons awareness that biomass or production, parameters typically measured in field studies to infer success of particular biota, are not directly acted upon by natural selection. Under coexistence with a competition specialist, a balance of competitive and defensive ability of the defense strategist was found to be evolutionarily stable, whereas stronger defense was optimal under increased nutrient levels in the absence of the pure competition specialist. The findings of success of different defense strategies are discussed with respect to SAR11, a highly successful bacterial clade in the pelagic ocean.

## Introduction

Partitioning of resources between competition and defense mechanisms is a dilemma relevant to members of almost any biological system, human societies and marine plankton communities included [1], [2]. In microbial ecology, both few-species [3], [4] and multispecies [5]–[7] experiments have provided ample evidence that coexistence on one limiting resource can be maintained top-down by selective loss factors such as size-selective predation or host-specific viral lysis. This has been termed the “Killing the Winner” (KtW) principle in microbial ecology [8], [9] and works through the selective loss mechanism preventing the fastest growing organism (competition specialist 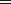 winner in the absence of selective loss) from exploiting all of the limiting resource, thereby leaving resources for the more slowly growing defense strategist. The same structure leading to top-down control of biodiversity also occurs in macro-ecological communities, where it is known under “Keystone Predation” (KP) [10]–[12].

**Figure 1:**
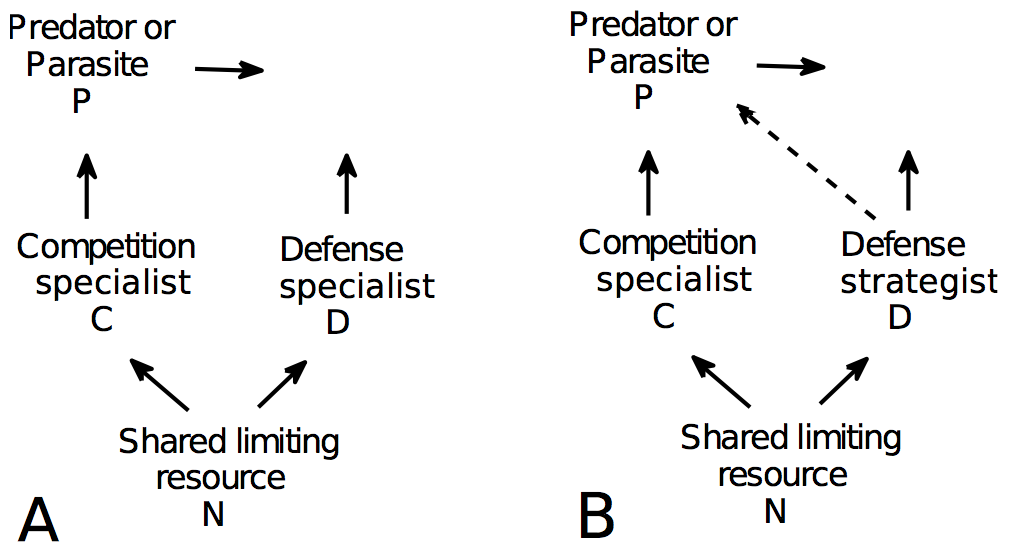
Killing-the-Winner (KtW) model with and without partial defense. Original KtW model with complete defense (no predation on defense specialist, A) and modified version with partial defense analyzed here (B). The mortality rate of the predator or parasite is indicated with a horizontal arrow. The total nutrient content in the system 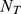 is the sum of N, C, D and P.

KtW generates a simple model (Figure 1A) with a potential to link a range of related microbiological phenomena including: the occurrence of grazing resistant forms of bacteria [6] and phytoplankton [5], [7], increasing phytoplankton cell size with increasing total-chlorophyll [13], the co-existence of apparently P-limited bacteria with P-limited phytoplankton [3], [14], [15], the occurrence of defense mechanisms against protozoan grazing in pathogenic bacteria [16], the genetic evidence that pelagic bacteria seem to be substrate generalists in terms of their ability to degrade organic material [17], and the coexistence of bacterial strains with different defense profiles against viruses [4]. All of these can be classified as variations over the classical theme of Hutchinson's Paradox [18] of an apparently smaller number of niches with respect to resources than coexisting phytoplankton species in the ocean, where top-down control imposes additional limiting factors to maintain diversity [10], [19].

In a modernized version, Hutchinson's Paradox could be extended to include the question of what maintains the huge biodiversity now observed in the prokaryote community, not only in terms of existing species, but also in terms of co-existing strains within these species [20]. A particularly interesting phenomenon related to the marine prokaryote community is the numerical dominance often found of SAR11 [21], leading to the question of whether this clade can be characterized as consisting of defense strategists [22] or efficient competitors [23], [24], and thus what determines the success of a particular strategy in the microbial part of the oceanic ecosystem. At present, the conceptual framework needed to address such questions seems relatively poorly developed, but see [25], [26].

Here, we extend the KtW concept to include partial defense, resulting in a diamond-shaped food web structure (Figure 1B) typically considered in classical KP analyses [19], [27]. Whereas a trade-off between competitive ability for resources and resistance to predation is typically assumed [4], [7], [27] and experimental evidence for it exists [11], [28], relatively few models of top-down control on coexistence and biodiversity contain so far a formalized representation of the trade-off [25], [26], [29]–[31]. In a virus-host community model [31], the effect of trade-off between competition and viral defense on the rank-abundance distributions of host strains and their associated viruses was studied. Here, we include a similar formalization of the trade-off between competition and defense in a simpler, generic three population food web model that consists of one predatory (P) and two competing prey populations (competition specialist C, and defense strategist D) (Figure 1). This gives us a framework to address questions on the success of particular strategies in both the microbial part of the oceanic ecosystem and other communities. In particular, we focus on one simple, but important aspect of the conceptual model: What characterizes the optimal defense strategy? We first study this by steady state analysis to determine which defense strategy corresponds to maximum biomass or production of the defense strategist. Then we compare these to the evolutionarily stable strategy (ESS), which is found through analysis of partial derivatives of the net growth rate with respect to the defense strategy. Defense strategies corresponding to maximum biomass, production or the net growth rate of invading individuals were found to be different and generally increased with increasing nutrient loads. A balance between competition and defense was found to be evolutionarily stable under coexistence of all three populations. A complex topology of the biomass distributions depending on the trade-off shape between competition and defense, the investment into defense, and the systems nutrient content indicate that understanding trade-offs mechanistically and quantitatively may be required for a better understanding of the success of particular strategies and the food web structure in aquatic microbial communities.

## Analysis

With the restricting assumption that food consumption is proportional to food concentration, which is a valid approximation under nutrient limitation found in oligotrophic regions of the pelagic ocean, the three-population KtW model can be given a Lotka-Volterra type formulation, simple enough to allow for an analytical solution for the equilibrium point (Table 1A). The analytically more complex situation with food consumption saturating at higher food levels can also be approached using graphical analysis [32]. In the simple linear version, there are three sets of parameters (Table 2) representing properties of the organisms (the nutrient affinity or clearance rate 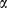, and the yield 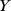), connections to higher trophic levels (the mortality rate 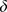), and properties of the environment (the total nutrient content 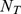). The system is assumed to be closed, such that mass balance is obtained (Table 1A, left). The system is analyzed at steady state by calculating the biomass of all three populations at equilibrium (i.e. 
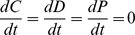
). An important property of the equilibrium point (Table 1A, right) is that biomass for the competition specialist (C*) and the predator (P*) are fixed functions of the 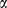, 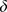 and 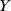 parameters, while equilibrium biomass of the defense strategist (D*) increases proportionally to the total amount of limiting nutrient 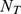. This simple model thus demonstrates a link between food web structure and resource conditions, suggesting a dominance of competition specialists in oligotrophic regions and a dominance of defense strategist in eutrophic (sensu total nutrient content) regions.

**Table 1:** Mass balance equations and equilibrium solutions for competition specialist (C), defense strategist (D), predator (P) and free nutrients (N) for original and modified KtW with partial defense.

A: Original KtW	
Mass balance equations:	Equilibrium solution (i.e. for 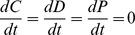 )
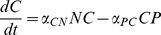	with C*, D*, and P* all 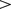 0:
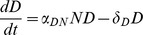	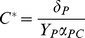
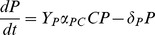	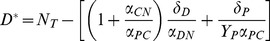
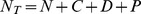	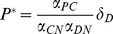

**Table 2:** Symbols and parameter values used including trade-off functions for defensive and competitive abilities of the defense strategist.

Name	Value	Description
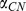	3	nutrient affinity for competition specialist
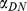	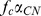	nutrient affinity for defense strategist
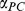	1	clearance rate of the predator on the competition specialist
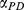	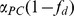	clearance rate of predator on defense strategist
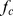	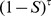	competitive ability of defense strategist
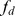	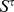	defensive ability of defense strategist
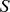	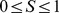	strategy index (0: pure competition, 1: pure defense)
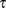	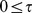	trade-off ( 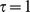 implies loss proportional to gain)
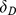	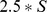 ; 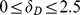	conditional loss rate of defense strategist to higher trophic levels
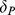	2.5	loss rate of predator to higher trophic levels
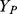	0.5	yield from predation
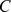 *		equilibrium solution for competition specialist
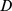 *		equilibrium solution for defense strategist
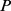 *		equilibrium solution for predator
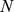 *		equilibrium solution for dissolved nutrients
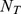		total nutrient content

Implicitly, this model assumes a trade-off between competition and defense. If an organism would be able to avoid paying a price in terms of a reduction in competitive ability when its grazing pressure or loss to viral lysis is reduced, then this organism would be able to monopolize all the resources. Theoretically, this is conceivable if an organism has for example found a means to use a non-limiting resource to reduce or remove the trade-off [33]. With no explicit representation of trade-off, a formalized analysis of the problem is, however, not possible with the original KtW model.

Defense mechanisms are numerous. In the pelagic microbial food web, they may include mechanisms such as changes in size or shape to avoid size-specific predators [34], [35], toxins [36], modification of surface properties [37] and/or intracellular defenses at the molecular level such as the CRISPR system [38]. The cost of different defense systems in terms of loss in growth rate is not immediately obvious and presumably quite variable. For a generic description, the trade-off should therefore be described with a parameter, the value of which can represent different mechanisms at the cellular level.

### Incorporating a formalized representation of trade-off

Theory shows that the shape of trade-off between two properties, i.e. whether costs tend to accelerate or decelerate with changing property, is as important as the magnitude of the costs [39]. In our model, the shape and magnitude of the trade-off can be modified through a single trade-off parameter 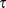. Following the formalized description of trade-off between competition and defense in a virus-host community [31], we represent trade-off relationships by introducing a strategy index 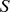 for the defense strategist, so that 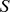 varies between 0 (pure competition specialist) and 1 (pure defense specialist). The strategy index 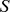 can then be converted into two indexes 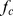 and 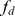, representing competitive and defensive abilities of the defense strategist, respectively:
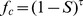


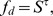
where the trade-off parameter 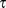 is a dimensionless positive number. These trade-off functions are used to relate the nutrient affinity of the defense strategist (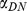) to that of the competition specialist (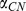) as



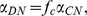
and the clearance rate of the predator on the defense strategist (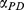) to its clearance rate on the competition specialist (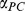) as



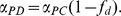



**Figure 2:**
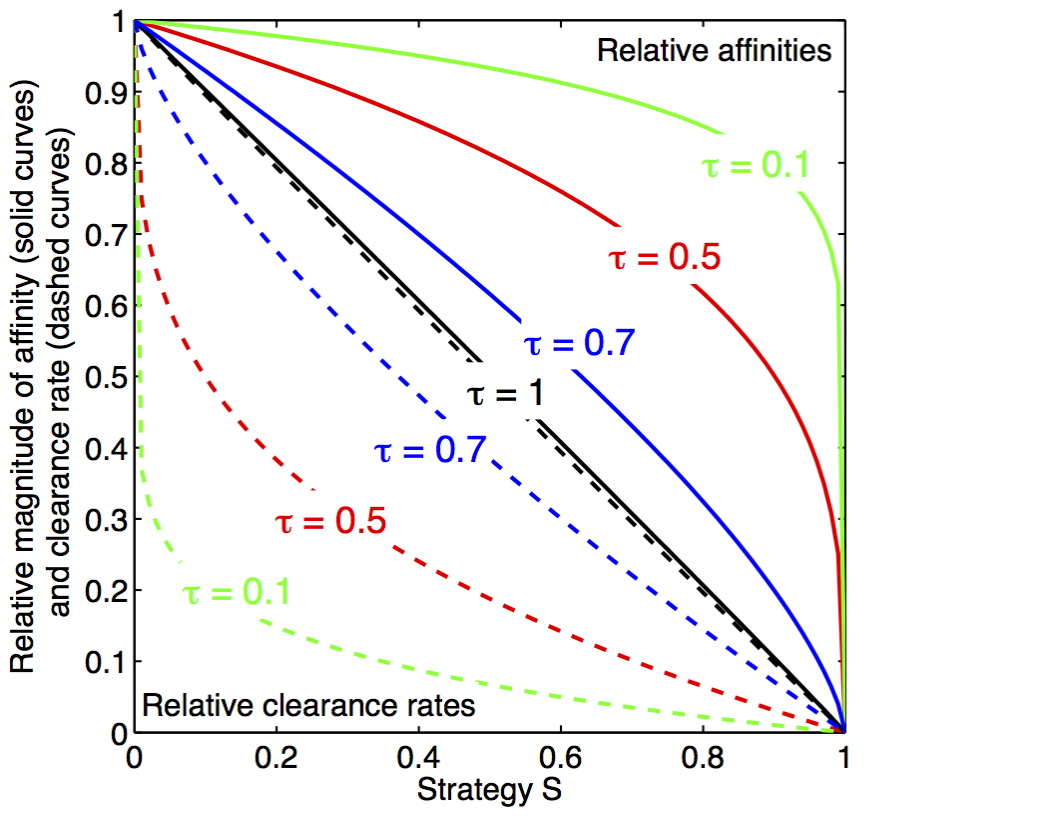
Trade-off functions between competitive and defensive abilities of the defense strategist. Relative affinity of defense strategist and clearance rate of predator on the defense strategist with respect to the defense strategy 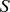 (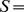
0 pure competition, 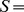
1 pure defense). For a trade-off parameter 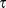 of 1 (dashed line), a linear trade-off shape is obtained where the loss in competitive ability (i.e. reduction of affinity of the defense strategist) is proportional to the gain in defense (i.e. the reduction of the predator's clearance rate) as the strategy 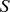 increases. For a trade-off parameter 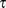 below 1 (solid lines, shown for 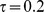), a trade-off is obtained where the clearance rate drops initially more steeply than the affinity for increasing 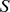, illustrating that a lot is gained initially in terms of reduced predation for a small reduction in competitive ability. The extension to a high trade-off parameters (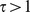) is trivial (i.e. the initial gain in defense is small relative to the loss in competition), but not of interest here since solutions with the defense strategist present only exist for 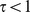 (not shown).

The nutrient affinity of the defense strategist relative to the competition specialist, and the predator's clearance rate on the defense strategist relative to the clearance rate on the competition specialist, are thus represented by the two functions 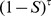 and 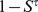, respectively (Figure 2). These two functions have the trade-off property that when the trade-off parameter is small (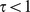), an initial loss in competitive ability (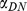) of the defense specialist for increasing 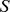 is small compared to the gain in defensive ability against the predator (i.e. inverse of clearance rate 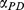). This situation corresponds to a convex trade-off function where resistance is increasingly costly [29], [40] and gives bended curves as shown in Figure 2. A linear trade-off function is obtained for 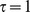, where the balanced situation occurs that a change in defense strategy 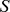 produces a loss in one property (e.g. defense ability) that is proportional to the gain in the other (e.g. competitive ability), and vise versa (straight line in Figure 2). For high trade-off parameters (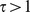), a modest initial increase in competitive ability would lead to drastic reduction in defensive abilities, corresponding to a concave trade-off function where resistance is decreasingly costly [29], [40].

Instead of the absolute defense specialist previously assumed in KtW models (Figure 1A), we are now in the position to introduce partial defense by using a defense strategy 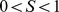 (Figure 1B). This means that the defense strategist can defend itself only partially against the predator or parasite (thus we avoid term “specialist” for the defense strategist). Hence, partial defense increases both the predator's clearance rate on the defense strategist (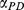) and the defense strategist' nutrient affinity (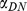) relative to complete defense.

Coexistence of all three populations (competition specialist, defense strategist, and predator) only occurs for convex trade-off functions, i.e. for 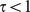. The differential equations and their equilibrium solution (obtained by setting 
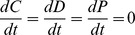
) for the case where the three populations coexist (all solutions C*, D*, and P* 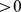) are shown in the upper half of Table 1B. When the solution for either C* or D* becomes negative, the system is reduced to a linear food chain with either D* or C*, respectively, as the remaining competitor and prey. These solutions are given at the lower half of Table 1B.

The stability of the solutions was tested using phase-plane plots. These plots show the evolution of the three populations (in terms of biomass) over time as described by the differential equations in Table 1. The solver ode45 from Matlab (version R2011s) was used to solve the differential equations. For varying initial population biomasses spanning orders of 10^0^ to 10^3^ and sets of parameters ranging from orders of 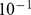 to 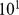, stable equilibria where populations remain constant over time were always reached. Equilibria obtained for mortality rates and predation yield on the order of 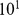 lead to different equilibria than those obtained for mortality rates and yield on the order of 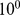 and lower (not shown).

### Finding the evolutionarily stable defense strategy

System equilibrium, as for example obtained in chemostat environments, does not necessarily imply evolutionary stagnation [41]. Even at stable populations size, slow growing individuals can be replaced through faster growing ones [42]. Evolutionary robustness of the strategies associated with maximum biomass and production was thus tested by comparing them to the evolutionarily stable strategy (ESS). The ESS is defined as the defense strategy 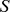 that cannot be invaded by mutants with slight deviations in 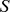. Hence, mutations away from the ESS imply a reduction of the defense strategist's net growth rate (Equation 1 in Appendix S1). Note that at steady state, the net growth rate of the defense strategist is zero per definition. The ESS is thus found by considering partial derivatives of the defense strategist's net growth rate with respect to the defense strategy 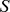 (see Appendix S1). The critical point where the first partial derivative (Equation 2 in Appendix S1) equals zero and the second partial derivative (Equation 3 in Appendix S1) is negative corresponds to a local maximum in the net growth rate. The strategy 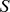 corresponding to the critical point is thus the ESS. With all three populations present, the ESS is found analytically to be 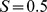 (Equation 4 in Appendix S1). With the competition specialist being absent, the solution for the ESS is more complicated (Equation 5 in Appendix S1) and found numerically by solving the first partial derivative of the net growth rate as a function of 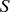, identifying the critical point where the function equals zero and confirming that the second partial derivative is negative at the critical point (see Appendix S1).

## Results

**Figure 3:**
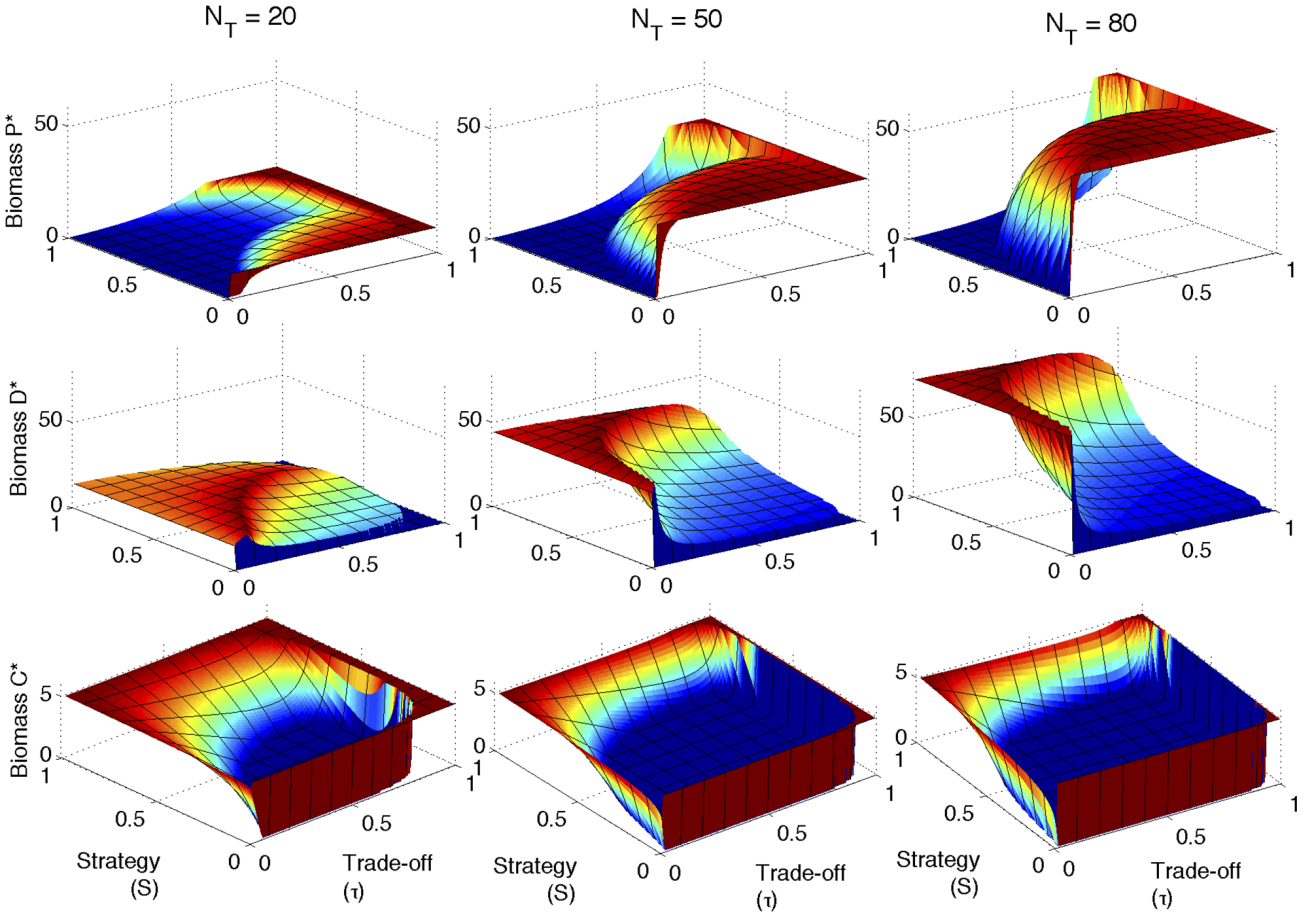
Biomass distributions at steady state as a function of defense strategy 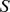 and trade-off parameter 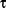. Steady-state biomass distributions for the predator (P*, top), the defense strategist (D*, middle) and the competition specialist (C*, bottom) with respect to the defense strategy 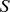 and trade-off parameter 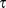 for three limiting nutrient contents (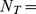
20, left, 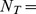
50, middle, and 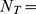
80, right). Other parameters as in Table 2.

The equilibrium solutions of the system as functions of the defense strategy 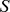 and trade-off parameter 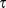 comprise three different regions in the 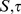 - parameter space: A) The competition specialist outcompetes the defense strategist, B) the defense strategist outcompetes the competition specialist, and C) competition specialist and defense strategist coexist. When the defense strategist is present, it is predominantly anti-correlated with the predator (Figure 3). The competition specialist outcompetes the defense strategist for all defense strategies when the trade-off parameter is close to 1. The defense strategist outcompetes the competition specialist when the trade-off parameter and defense strategy are both either low or intermediate. Coexistence occurs predominantly for intermediate to high defense strategies at low to intermediate trade-off parameters.

The 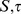 - region where the defense strategist outcompetes the competition specialist expands as the total nutrient content 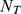 of the system increases (Figure 3, left to right). Consequently, while the maximum population size of the competition specialist is independent of resource level, as in the original KtW model (Table 1A), the competition specialist is more vulnerable at high 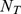 because the set of 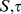-combinations where it can coexist with the defense strategist decreases. The predator population, which was independent of 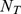 in the original KtW model, now increases with 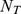, since it also gains resources through partial predation on the defense strategist (Figure 3, top). When 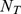 is sufficiently low, there is no 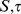 -pair for which the competition specialist is outcompeted (not shown).

Interestingly, the transition from a three-population (C*, D*, and P*) to a two-population (D* and P*) community in the 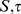 - region coincides with a maximum population size of the defense strategist. This maximum defines a sharp ridge, such that only slight deviations from the 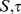 - pairs defining the maximum biomass result in a rapid drop of biomass. Biomass loss of the defense strategist for reduced 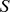 is reflected in the increased biomass of the predator. The strategy corresponding to maximum biomass changes from low defense at low trade-off parameters to intermediate defense at intermediate trade-off parameters, whereas it tends towards less defense again for trade-off parameters approaching 1.

**Figure 4:**
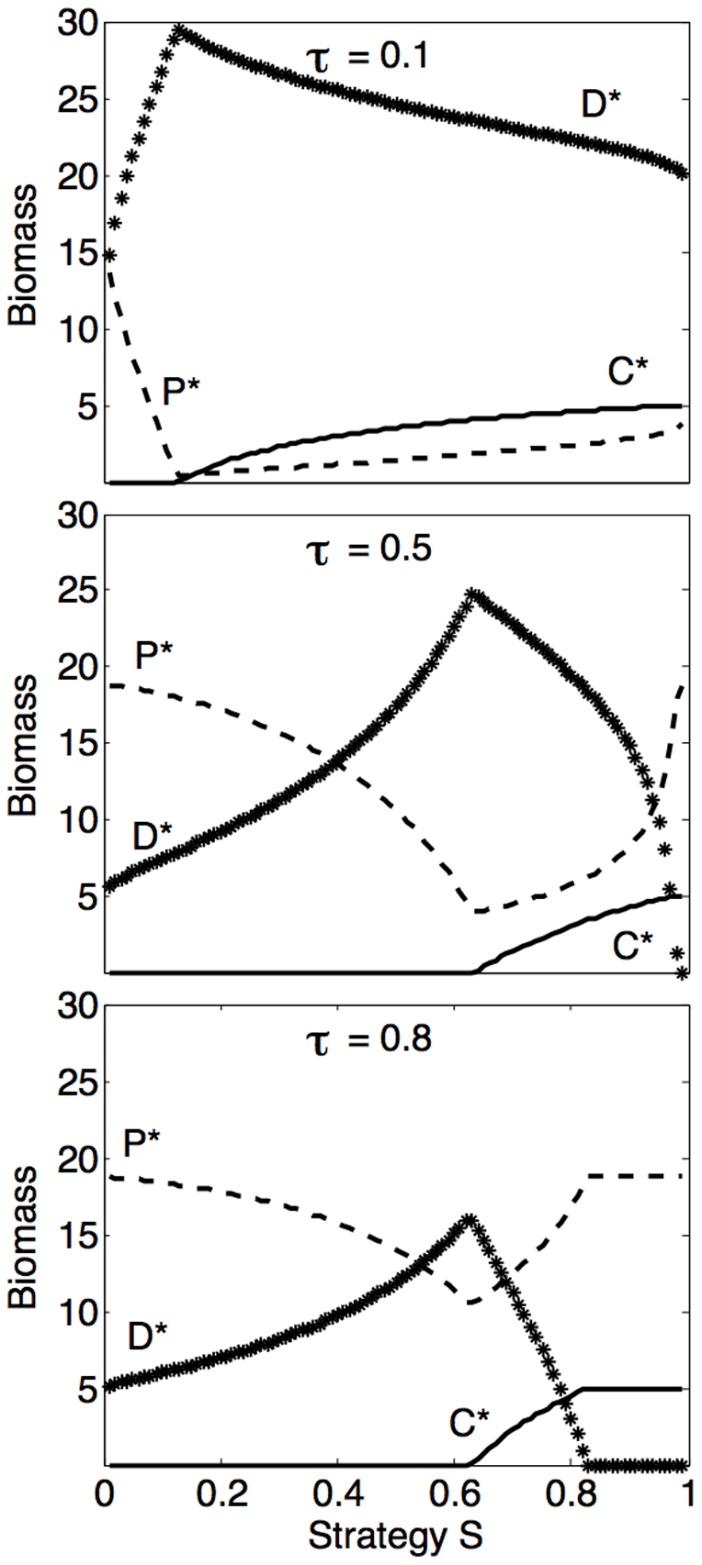
Biomass sections as a function of the defense strategy 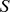 for given trade-off parameters 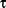. Steady-state biomass of competition specialist (C*, fine dotted lines), defense strategist (D*, dashed line) and predator (P*, solid line) as a function of defense strategy for different trade-offs (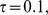 top, 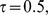 middle, and 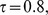 bottom) for 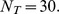 Other parameters as in Table 2.

Figure 4 shows the response of the equilibrium C*, D*, and P* populations to changes in the defense strategy 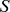 for given fixed trade-off parameters (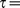
0.1, 0.5 and 0.8 at 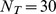), illustrating the anti-correlation of P* (dashed line) and D* (thick starred line). Also, the narrow range of defense strategies resulting in a sharp biomass maximum of the defense strategist is clearly visible as a peak in the thick starred lines of Figure 4.

**Figure 5:**
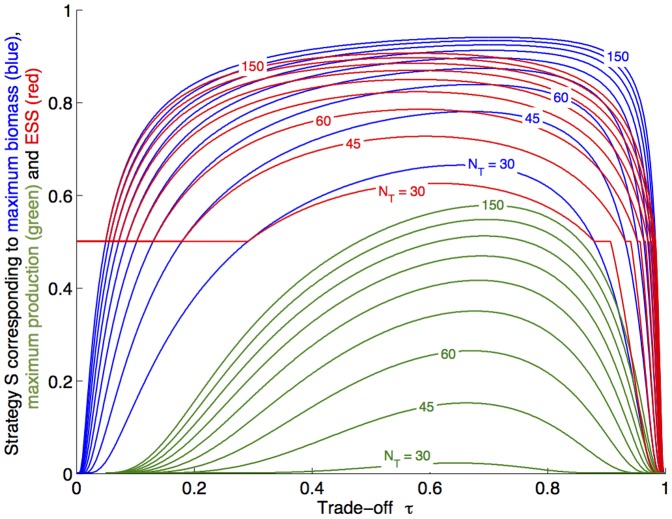
Optimal defense strategies with respect to maximum biomass, maximum production and evolutionarily stable strategy (ESS). Defense strategies 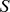 corresponding to defense strategist's maximum biomass (blue), maximum production (defined as 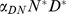, green) and ESS (red) are shown as a function of the trade-off parameter 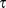 for different nutrient contents. The ESS is defined by the maximum net growth rate of a invading mutant, which is found by critical point analysis of the first partial derivative of the net growth rate with respect to strategy 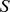 (see Appendix S1). Different contours show the effect of the total nutrient content 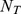 on the maximizing strategies. Other parameters as in Table 2.

The defense strategy corresponding to maximum biomass of the defense specialist depends on the total nutrient content of the system and is shown as a function of the trade-off parameter 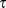 in Figure 5 (blue contours). At high nutrient contents, the defense strategy corresponding to maximum biomass is higher than at low nutrient contents. Also, at high nutrient contents, the strategy corresponding to maximum biomass remains similar over a wide range of the trade-off parameter 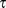, whereas it varies more as a function of 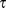 at low nutrient contents.

The defense strategy corresponding to maximum production of the defense strategist (defined as gross growth rate 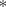 biomass, i.e. 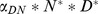) as a function of the trade-off parameter 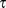 differs from that corresponding to maximum biomass (Figure 5, green contours). The most marked difference is that maximum production generally requires a lower defense strategy (Figure 5, blue vs green contours).

The defense strategy corresponding to the maximum net growth rate of invading mutants (defined as the ESS, which is found by critical point analysis where the first partial derivative of the net growth rate with respect to 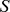 equals zero and the second partial derivative is negative, see Appendix S1), is generally different from the strategies corresponding to maximum biomass or production (Figure 5, red contours). When all three populations are present, the ESS is 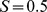 (resulting in horizontal red lines in Figure 5). The region where the competition specialist coexists with the defense strategist decreases for increasing 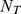, causing the horizontal red lines to be narrower at high 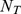. When the competition specialist is outcompeted by the defense strategist, the ESS varies as a function of the trade-off parameter and total nutrient content (bended red contours in Figure 5). In this case, the ESS increases with increasing nutrient content, and generally resembles the strategy corresponding to maximum biomass (comparing red and blue contours in Figure 5).

## Discussion

Even though trade-offs between competitive and defensive traits are often assumed, modeling studies on the effect of top-down control on coexistence and biodiversity traditionally lacked a formalized representation of trade-offs [8], [12], [19]. More recently, evolutionary models have been used to study the influence of adaptive change on the success of strategies linked to a trade-off between defensive and competitive abilities [25], [26]. In contrast to modeling studies with trade-off functions focusing on stability analysis of the steady state solutions [29], [40], or studies where trade-offs between competition and defense were modeled specifically based on biophysical constrains related to cell size [43], [44], the main goal of this study was to construct a generic model framework that allows us to analyze the interplay of competition, predation and partial defense under the influence of different trade-offs and nutrient contents in a simplified food web. As we are interested in the microbial part of the pelagic ecosystem, we discuss our result in light of the KtW model, indicating implicitly that the results relate to the KP concept as well. Hence, the presented analyses draws the attention to a connection between macro- and microbiological theory, which has previously been obscure.

Although based on the mathematically relatively simple KtW model, we found that the steady state solution has a complicated topology depending on the trade-off parameter, the partial defense strategy and the systems nutrients content. Some of the complexity in the behavior of the present model is rooted in the compensating mechanism inherent in partial defense. By partially stimulating the predator, the consequence of the defense strategist is reduced population size of its competitor. This leads to “apparent competition” between the two competitors [27], [45], and has some resemblance to the situation where a mixotroph can get a double bonus from eating its competitors by not only sequestering the mass and energy contained in the prey, but also removing a nutrient competitor [46]. Note, however, that in contrast to “apparent competition”, “eating your competitor” [46] involves direct interaction of the competitors.

### Trade-offs in pelagic microbial ecosystems

The relationships between competition and defense strategies investigated here are central in a variety of ecological studies. Although it is difficult to identify and quantify trade-offs between defense and competition in natural microbial food webs, trade-offs are increasingly recognized as central in modeling marine microbial communities [47]. Algae that are edible for *Daphnia* tend to have higher nutrient affinities than indelible ones, suggesting that edible algae are better competitors [11]. A rotifer species with supposedly low defense abilities was found to reach higher densities at low resource levels than a superior defense specialist [48], whereas high resource densities favored the growth of the defense specialist in qualitative accordance with our model. Also, *E. coli* strains being partially resistant to phages seem more successful at high nutrient concentrations than sensitive strains [4]. These findings match our model predictions that competition is a stronger selective force at low nutrient concentrations, while predation controls the community structure at high resources. However, experiments with terrestrial plants [49] suggest that synergistic effects can potentially occur between defense and competition strategies. Whether this means that predation and parasitism is a stronger selective force in marine microbial systems than in terrestrial plant communities remains open. Certainly, further efforts need to be made to understand trade-offs in microbial communities on a mechanistic level.

Trade-offs between competition and defense arising from biophysical constrains related to body size are widely accepted for microbial communities and have been studied in size-structured models of planktonic ecosystems [43], [44], [50]. Our general finding that high nutrient content leads to higher biomass of the defense strategist is consistent with the specific findings that increased nutrient contents gives the potential for larger (more defensive) size classes to establish, given that grazers control the smaller (more competitive) size classes [43], [44], [50].

### Linking strategy choice to trade-off and biogeochemistry

An important conceptual aspect of the original KtW-model, also described for the KP concept [12], [51], is its coupling of food web structure to biogeochemistry, where oligotrophic systems (low 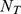) are dominated by the predator-controlled competition specialists, while eutrophic ones (high 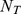) become progressively dominated by resource controlled defense specialists [8], [52]. The somewhat counter-intuitive property of a maximum population size of the competition specialist independent of resource level is carried over to this extended model with partial defense and trade-off. Here, however, increasing resource levels also expands the set of 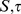 -values where the defense strategist can exclude the competition specialist from the system, suggesting that pure competition strategies are increasingly vulnerable at high 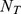. Together with the trend for higher optimal defense strategies (with respect to maximum biomass, production and ESS) for high nutrient contents (Figure 5), this is in line with a selection experiment of moths and viruses, where high resistance evolved more easily when resource levels were high [39].

There is an interesting parallel in zoological marine ecology to the general trend of increased emphasis on defense with increasing resource level (Figure 5). While one could assume that an increase in food resources to zooplankton or fish larvae should lead to increased growth, several models of optimal behavior [53], [54] show that organisms should rather migrate downwards to less illuminated waters where mortality risk from visual predators is lower [55]. This also explains the common observation in field studies that growth rate is seemingly food-independent [56], [57].

Evolutionary dynamics may change effects of nutrient enrichment on food web structures as predicted by classical steady-state models. For instance, when plants alone were allowed to evolve in an adaptive nutrient-plant-herbivore food chain model, increased nutrient contents could lead to higher biomass of both the plant and herbivore population [26], whereas evolution in both the plant and herbivore population or the herbivore population alone typically resulted in nutrient-independent biomass of the plant population, in agreement with the present steady-state analysis when considering the plant as the competition specialist. However, the outcome of plant and herbivore biomass also depended on the shape of the trade-off [26], also consistent with our study.

The discussion above links defense strategy to biogeochemical cycling. This is illustrated by the extreme example of a community of defense strategists being numerically abundant, but not processing significant amounts of material and energy. The fluxes of energy and material in the system are dominated by the populations dominating in production, not in abundance. A review on bacterial standing stocks and production suggests that standing stocks are similar throughout the euphotic zone, while production varies more widely [58]. This indicates that bacterial production is to some extent regulated independently from biomass, which is well known for primary production, where phytoplankton contribute to roughly 50% of the global net primary production, but only make up a small fraction of the standing stock of photosynthetic biomass on Earth [59].

### What are successful strategies in the pelagic ocean?

The model illustrates how different defense strategies may lead to maximum biomass or production. In particular, high defense is required in our model to obtain high biomass, whereas lower defense corresponds to higher production. One could argue that a high production increases the number of mutations produced per time unit and therefore enhances genetic flexibility, potentially reducing the need of heavy investment in defense. However, it is important to keep in mind that our analysis revealed strategies corresponding to maximum biomass or production that are not necessarily evolutionarily stable strategies. Biomass and production, quantities typically measured in field surveys, are thus not directly acted upon by natural selection, which should be considered when interpreting biomass and production as measures of success of particular biota. In the presence of both competitors, the evolutionarily stable strategy is 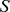 = 0.5, indicating that a strong balance between competitive and defensive abilities is optimal when both top-down (predation) and bottom-up (resource competition) control act on the defense strategist. Interestingly, 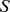 = 0.5 is evolutionarily stable both at low nutrient content (where bottom-up control is the dominant selective force) and at high nutrient content (where top-down control is predominantly controlling food web structure) [44], [60]. This matches with theoretical predictions of maximized fitness of any evolutionary unit when the investment into survival (in our case defense) and reproduction (in our case competition allowing faster growth) is equal [61], [62]. In the absence of the competition specialist, higher defense (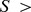
0.5) is evolutionarily stable. Also, instead of making use of excessive nutrients by growing faster, an increased defense is evolutionarily stable under increasing nutrient contents (Figure 5), in line with model predictions of increased survival but not increased growth under higher food availability [54].

The general framework of our model could have a potential to describe central aspects of biodiversity within communities such as e.g. heterotrophic prokaryotes. At present, the observational side of marine microbial ecology is a set of findings that are intuitively related, but not yet well-explained. A highly topical discussion relevant for the functioning of the pelagic microbial ecosystem is the general success (in terms of numerical dominance) of the SAR11 clade [63], a group of bacteria belonging to the Alphaproteobacteria [64]. The SAR11 clade reportedly represents between 25–45% of all microbial cells in the euphotic zone, and between 15–30% in the aphotic zone of oceanic regions [21], [65], [66]. It is an intriguing question whether this dominance is related to specific properties of the marine pelagic such as e.g. relative temporal and spatial homogeneity compared to other environments, and if so, whether SAR11 is successful under these conditions as a competition or as a defense specialist [24], [63]. According to our model, a high biomass is obtained through a high defense strategy, whereas lower defense strategies correspond to high production (Figure 5). The high numerical abundance of SAR11 [66], despite previously observed slow growth rates [67], would thus be consistent with our model if the majority of SAR11 strains have a high defense strategy (i.e. 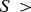
0.5).

Metagenome analysis has revealed islands in the genome of SAR11 strains that might be phage recognition sites used in effective viral defense [68]. A predominantly defensive strategy would also fit with observations of SAR11 cells growing slowly in nature [22], which does not change with increased nutrient concentrations [64], [69]. As circumstantial evidence, this could be consistent with a pelagic prokaryote community being dominated by defense specialists. Removing viral pressure from this community would then lead to a total population shift, where previously rare, but fast growing, competition specialists become dominating. Such shifts in community structure have been observed in experiments where viral pressure is reduced [70], and has recently also been shown in a global size-structured ecosystem model where removal of the KtW mechanism led to a widespread dominance of small competition specialists [71]. The recent discovery of abundant SAR11 viruses, however, has been used as an argument against the hypothesis of most SAR11 being defense specialists [24]. The small size and streamlined genome of SAR11 as well as their relatively high abundance in oligotrophic regions have already previously be interpreted as competitive traits [23], and field experiments revealed growth rates of SAR11 spanning the entire spectrum of other prokaryotes present in the sample. This suggests that at least some SAR11 strains are capable of fast growth and are thus competition specialists [72], supporting the idea that SAR11 is a group of highly diverse strains [63], [73], [74]. Interestingly, if SAR11 is a group consisting of both competitive and defensive strains indeed, the finding of abundant SAR11 viruses [24] fits with the KtW model predictions that the biomass of SAR11 viruses should be high when differences in growth rates of the coexisting SAR11 strains are large [75]. This is due to compensation of fast growth rates of the strains “winning” with respect to competition for limiting resources by increased viral infection at steady state [52].

### Diversity generating mechanisms

Whereas temporal and spatial heterogeneity in environmental conditions can increase biodiversity [76], [77], the KtW-model predicts coexistence also at steady state. Hence it adds to the understanding of the paradox of plankton [18], including prokaryotes. Being based on steady state arguments, our analysis represents a counterpart to the intermediate disturbance hypothesis [78], which focuses on fluctuations and their effects on biodiversity. There, r strategists dominate in frequently disturbed and K-strategists in stable environments, while maximum diversity is obtained at intermediate disturbance levels, where K and r strategists coexist. Generalizing the steady state version of KtW arguments, low nutrient systems should be dominated by competition strategists, nutrient rich systems by defense strategists, and the evenness component of diversity would be expected to be highest at intermediate nutrients level, where none of the two strategies dominate. This is in agreement with one of the earliest models on top-down control, where coexistence of two competitors most likely occurs at intermediate productivity [79].

### Model assumptions

Extreme defense-specialists cannot survive in our model since the competitive ability 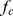, and thereby the clearance rate for defense strategist (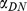), becomes zero for 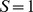. In nature, 100% effective defense strategies against selected loss mechanisms may be viable. For example, the CRISPR system in prokaryotes supposedly allows the host to be 100% resistant against those viruses that are incorporated in the genetic defense library [38]. On the other hand, even virus-resistant hosts can be eaten by predators, making 100% defense against all loss mechanism impossible.

Being a conceptual model, the absolute magnitude of the affinities and loss rates is secondary for our study. Important is the relative size of the parameters for the competition and defense specialists, modified by the trade-off functions 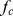 and 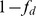 (Figure 2). Hence, the model can be applied to both microbial food webs and other top-down controlled communities.

The loss rate 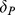 is considered constant in our analysis. Since this can be seen as parametric representation of the loss to higher trophic levels, it would in a more complex food web setting depend on the biomass at the next trophic level. Consequently, there would be a connection between 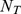 and the 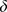 -value that we have not attempted to include here. Among the two competing populations C and D, a loss rate to higher trophic levels (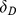) was modeled only for the defense but not competition specialist. We justify this choice by considering natural food webs. When organisms avoid predation on one trophic level (in our model considered defense specialists), they are still predated upon by higher trophic levels. As an example, we can consider large, single-celled algae as defense specialists avoiding predation by heterotrophic nanoflagellates or bacteriophages (corresponding to P), but algae still experience loss to higher trophic levels including micro- and mesozooplankton or algal viruses (modeled by 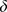 D). This contrasts bacteria (here corresponding to C), which are better competitors than the algae but die predominantly through grazing by heterotrophic nanoflagellates or infection by bacteriophages (P). The loss rate of D to higher trophic levels (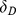) is scaled with the defense strategy 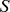 such that when D has the strategy 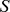 = 0 (i.e. D is a pure competition specialist), then D experiences the same loss as C (i.e. no additional loss to higher trophic levels) (Table 2).

The parameterization of competition for one generic resource only is a simplification allowing for the intended generic analysis of trade-off consequences for the food web structure. Hypothetically, one could imagine models of more complex food webs, where all trophic interactions are represented by trade-offs. This would link ecosystem models closer to the constraints experienced at the level of individuals in nature, and attempts in this direction has indeed given interesting results [80]. However, combining our present lack of qualitative and quantitative knowledge on trade-offs between strategies with the complexity of the solutions suggested by the present analysis, food web models with extensive detail in trade-off descriptions may seem difficult to achieve. Further experimental effort needs to be made to quantitatively understand trade-offs and the underlying mechanisms associated with defense.

## Conclusions

The extended KtW-model with partial defense and a formalized representation of trade-off between competition and defense allowed us to study links between food web structure, defense strategies, trade-offs and nutrient contents in the system. In systems with high nutrient contents, high defense was favorable for both biomass and production, although defense strategies corresponding to maximum biomass were consistently higher than those corresponding to maximum production. Importantly, strategies corresponding to maximum biomass or production were found to not necessarily be evolutionarily stable. Under coexistence of both competitors, when top-down and bottom up control act on the defense strategist, a balance between competitive and defensive abilities was found to be evolutionarily stable, whereas increasing defense was evolutionarily stable at increasing nutrient loads when the competition specialist was outcompeted. In the latter case, the ESS varied as a function of the trade-off parameter and resembled the strategy corresponding to maximum biomass. Despite the highly simplified food web structure presented here, the analysis may help understand the success of SAR11 in terms of numerically dominant strains with strong defensive abilities.

Trade-offs between strategy choice appear central in biology, but the theoretical framework to study trade-off in ecosystems is relatively poorly developed so far. While experimental studies are necessary to identify and quantify specific trade-off mechanisms that are fundamental to understanding the success of particular biota such as the SAR11 clade, finding in general that trade-off strongly influences the food web structure as shown in this study should summon ecological modelers to further develop representations of trade-offs in future, larger scale ecosystem models. Also, illuminating parallels between the concepts of KP and KtW based on similar ecological processes in macro- and microbiological systems, the work should stimulate further development of unifying principles across different biological disciplines.

## Supporting Information

Figure S1
**Critical point analysis for the defense strategists net growth rate.** The defense strategist's net growth rate (green), the first partial derivative of the defense strategist's net growth rate (black) and the second partial derivative of the defense strategists net growth rate (red) with respect to 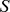 are plotted as a function of 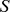 for different trade-off parameters 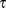 at a total nutrient content of 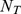 = 150.(TIFF)

Appendix S1 **Finding the evolutionarily stable strategy (ESS).** The appendix S1 shows how the evolutionarily stable strategy (ESS) of the defense strategist is found analytically and numerically in the presence of all three populations and in the absence of the competition specialist, respectively.
(PDF)
