# Supplementary figures and images for: Optimal Defense Strategies in an Idealized Microbial Food Web under Trade-Off between Competition and Defense
Source: PLoS One. 2014 Jul 7;9(7):e101415. doi: 10.1371/journal.pone.0101415 (PMC4084851; doi:10.1371/journal.pone.0101415)

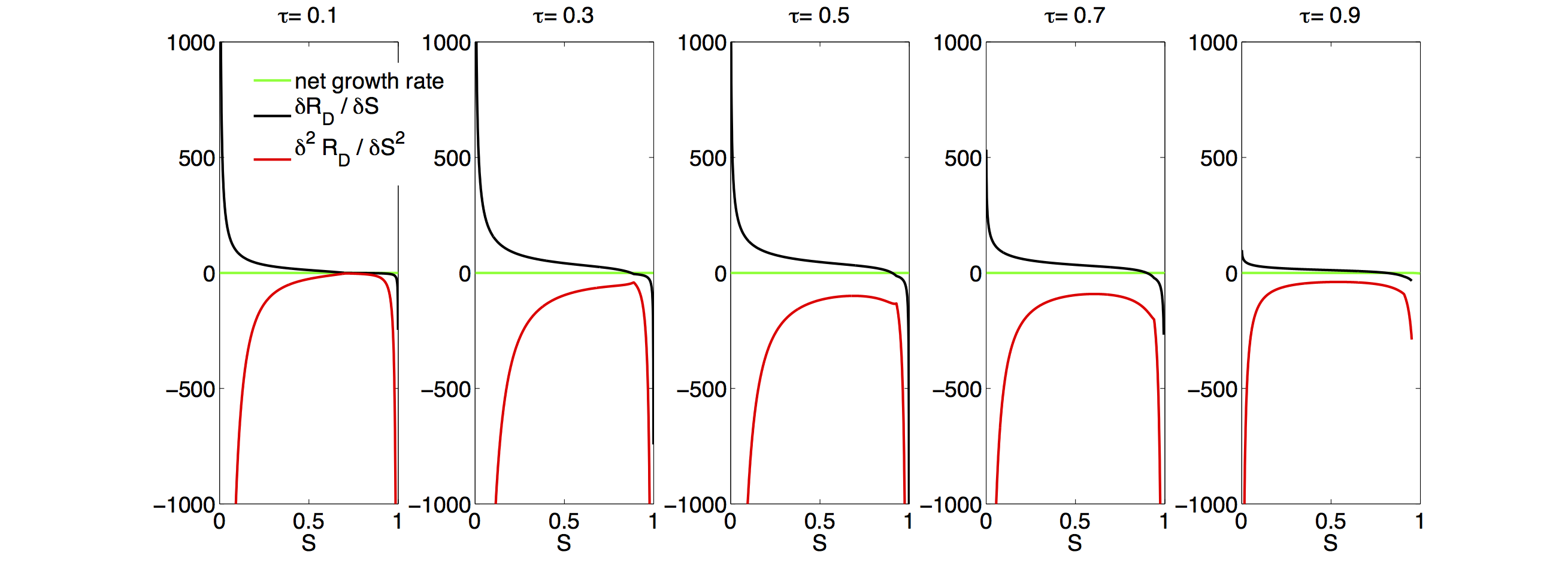

Supplement: Figure S1 — Critical point analysis for the defense strategists net growth rate. The defense strategist's net growth rate (green), the first partial derivative of the defense strategist's net growth rate (black) and the second partial derivative of the defense strategists net growth rate (red) with respect to are plotted as a function of for different trade-off parameters at a total nutrient content of = 150. (TIFF) [file pone.0101415.s001.tiff]
