# Supplementary material for: Optimal Defense Strategies in an Idealized Microbial Food Web under Trade-Off between Competition and Defense
Source: PLoS One. 2014 Jul 7;9(7):e101415. doi: 10.1371/journal.pone.0101415 (PMC4084851; doi:10.1371/journal.pone.0101415)
Supplement: Appendix S1 — Finding the evolutionarily stable strategy (ESS). The appendix S1 shows how the evolutionarily stable strategy (ESS) of the defense strategist is found analytically and numerically in the presence of all three populations and in the absence of the competition specialist, respectively. (PDF) [file pone.0101415.s002.pdf]

Appendix S1

To find the evolutionarily stable strategy (ESS) of the defense strategist, we consider the defense strategist's net growth rate R_D (from Table 1B in article):

$$R_D = \alpha_{DN} N^* - \alpha_{PD} P^* - \delta_D = (1-S)^\tau \alpha_{CN} N^* - (1 - S^\tau)\alpha_{PC} P^* - \delta_D \quad (1)$$

The first partial derivative of R_D with respect to S (i.e. $\frac{\partial R_D}{\partial S}$) is:

$$\frac{\partial}{\partial S}[(1-S)^\tau \alpha_{CN} N^* - (1-S^\tau)\alpha_{PC} P^* - \delta_D] = -\tau(1-S)^{\tau-1}\alpha_{CN} N^* + \tau S^{\tau-1}\alpha_{PC} P^* \quad (2)$$

The second partial derivative of R_D with respect to S (i.e. $\frac{\partial^2 R_D}{\partial S^2}$) is:

$$\frac{\partial}{\partial S}[-\tau(1-S)^{\tau-1}\alpha_{CN} N^* + \tau S^{\tau-1}\alpha_{PC} P^*] = \tau(\tau-1)(1-S)^{\tau-2}\alpha_{CN} N^* + \tau(\tau-1) S^{\tau-2}\alpha_{PC} P^* \quad (3)$$

The ESS for the defense strategist when all three populations (C, D, and P) are present is found analytically by setting the first partial derivative of the net growth rate R_D with respect to S equal to zero and solving for S. For $\tau > 0$, using the equilibrium solution $N^* = \frac{\alpha_{PC}}{\alpha_{CN}} P^*$ (Table 1B in article) when all three populations are present, this gives an ESS of S = 0.5, independent of the parameter values used:

$$\frac{\partial R_D}{\partial S} = -\tau(1-S)^{\tau-1}\alpha_{CN} N^* + \tau S^{\tau-1}\alpha_{PC} P^* = 0$$

$$\frac{1-S}{S} = \left(\frac{\alpha_{PC} P^*}{\alpha_{CN} N^*}\right)^{\frac{1}{\tau-1}} = \left(\frac{\alpha_{PC} P^* \alpha_{CN}}{\alpha_{CN}\alpha_{PC} P^*}\right)^{\frac{1}{\tau-1}} = 1$$

$$\Rightarrow \quad S = 0.5 \quad (4)$$

The analytical solution for the ESS when the competition specialist is absent is more complicated and varies as a function of the trade-off parameter τ and the total nutrient content N_T. Using the equilibrium solution $N^* = \frac{\alpha_{PD} * P^* + \delta_D}{\alpha_{DN}}$ (Table 1B in article) when C is absent, we get:

$$\frac{1-S}{S} = \left(\frac{\alpha_{PC} P^*}{\alpha_{CN} N^*}\right)^{\frac{1}{\tau-1}} = \left[\frac{\alpha_{PC}}{\alpha_{CN}} P^* \frac{\alpha_{DN}}{\alpha_{PD} P^* + \delta_D}\right]^{\frac{1}{\tau-1}}$$

$$\Rightarrow \quad S = \frac{1}{\left[\frac{\alpha_{PC}}{\alpha_{CN}} P^* \frac{\alpha_{DN}}{\alpha_{PD} P^* + \delta_D}\right]^{\frac{1}{\tau - 1}} + 1} \tag{5}$$

where $P^* = \dfrac{1}{1 + \alpha_{PD}/\alpha_{DN}} \left(N_T - \dfrac{\delta_P}{Y_P \alpha_{PD}} - \dfrac{\delta_D}{\alpha_{DN}} \right)$ (from Table 1B in article).

The ESS is thus found numerically instead by solving the first partial derivative of R_D with respect to S as a function of S for different trade-off parameters and total nutrient contents. Extracting the S-value for which the partial derivative equals zero gives the ESS. The second partial derivative with respect to S was calculated and found to be negative, verifying that the extremum where the first partial derivative equals zero is a local maximum. Graphically, this procedure is illustrated in Figure S1. Figure 5 in the article shows the ESS in red as a function of the trade-off parameter τ and different total nutrient contents N_T.
